# A Scoping Review of Emerging Treatments in the Pipeline for Idiopathic Pulmonary Fibrosis: Future Perspectives

**DOI:** 10.3390/biomedicines14061293

**Published:** 2026-06-05

**Authors:** Maria Eugenia Novara, Martina Chirulli, Patrizio Vitulo, Anna Carollo, Alessio Provenzani

**Affiliations:** 1 Mediterranean Institute for Transplantation and Advanced Specialized Therapies (IRCCS ISMETT), 90127 Palermo, Italy; pvitulo@ismett.edu (P.V.); ccarollo@ismett.edu (A.C.); aprovenzani@ismett.edu (A.P.); 2 Department of Biological, Chemical and Pharmaceutical Sciences and Technologies (STEBICEF), School of Specialization in Hospital Pharmacy, University of Palermo, 90133 Palermo, Italy; martinachirulli@gmail.com; 3 University of Pittsburgh Medical Center (UPMC) Italy, 90133 Palermo, Italy

**Keywords:** idiopathic pulmonary fibrosis, scoping review, pipeline, novel treatments

## Abstract

**Background**: Idiopathic pulmonary fibrosis (IPF) is an incurable disease with limited therapeutic options and a poor prognosis. Current standard therapies are characterized by drugs or surgical strategies with limited effects, as they are either not curative or their use is restricted to a specific subset of the population. The aim of this scoping review is to evaluate the drugs currently under investigation in Phase II and Phase III trials and provide an overview of the mechanisms of new therapeutic strategies for IPF. **Methods**: The search strategy was conducted in accordance with PRISMA guidelines and included studies conducted on adults with IPF retrieved from the registered ClinicalTrials.gov database up to 31 December 2025. **Results**: Nineteen studies were included. The clinical trials investigate key signaling pathways and molecular targets, including MAPK, RhoA/ROCK, PDE4B/cAMP, Wnt/β-catenin, Hedgehog/SMO, IL-11/STAT3, and LPA/autotaxin, as well as extracellular receptors and mediators such as CSF1R, TBXA2R, WISP1 and S100A4. **Conclusions**: Ongoing clinical research in IPF reflects a broad diversification of molecular targets; however, translational success remains limited. Current evidence suggests that biological complexity, pathway redundancy, and systemic constraints significantly restrict the clinical impact of single-target strategies. Future progress will likely depend on improved patient stratification, combination approaches, and biomarker-guided trial design rather than isolated pathway modulation.

## 1. Introduction

Idiopathic pulmonary fibrosis (IPF) is a chronic, progressive, fibrotic disease characterized by scarring and architectural distortion that causes a reduction in the lung’s respiratory ability and represents the most common form of pulmonary fibrosis (PF). The incidence of IPF is 3–9 cases per 100,000 person-years in Europe and North America, but appears to be lower in Asia and South America [1]. The causes of IPF remain unknown, although it is believed to arise from a combination of genetic and environmental factors [2,3]. IPF is thought to result from epithelial dysfunction, in which repeated injury to the lung epithelium causes defective repair mechanisms, leading to fibroblast activation, myofibroblast differentiation, and excessive extracellular matrix (ECM) deposition, resulting in chronic fibroproliferation. Multiple interconnected signaling pathways and molecular regulators contribute to disease pathogenesis, converging on epithelial injury, aberrant matrix remodeling, and dysregulated immune responses, thereby perpetuating the fibrotic process. Moreover, pulmonary hypertension is a common comorbidity in patients with IPF and is associated with a worse prognosis [4]. Pirfenidone and nintedanib are two approved antifibrotic therapies that slow lung function decline and modulate pathogenic pathways. However, they do not cure the disease, and their use may be limited by side effects requiring dose reduction or treatment discontinuation, including nausea, diarrhea, loss of appetite, and liver function abnormalities [5]. The only treatment with proven benefits is lung transplantation (LTx), which is considered only for a select subset of patients whose expected post-transplant survival exceeds their projected survival without transplantation [6,7]. Transplant evaluation is based on a multidisciplinary assessment that requires careful consideration of clinical and prognostic parameters [8,9,10]. Due to the limited efficacy of current pharmacological and non-pharmacological treatments, as well as the poor prognosis of the disease, it is important to examine current research directions and therapeutic strategies under investigation [11]. This scoping review aims to systematically map ongoing Phase II and III clinical trials and provide a mechanistic interpretation of the evolving therapeutic landscape in IPF.

### 1.1. Therapeutic Targets and Pathways Implicated in Pulmonary Fibrosis

Several signaling pathways and molecular regulators are implicated in the pathogenesis of IPF. These pathways converge on fibroblast activation, epithelial injury, macrophage polarization, and ECM remodeling, thereby perpetuating the fibrotic process.

The increased deposition of ECM proteins and the resulting pulmonary remodeling in PF are driven by the proliferation/apoptosis of fibroblasts and myofibroblasts and by the synthesis/degradation of ECM components. These processes lead to the loss of pulmonary elasticity and impaired gas exchange [12,13].

In this pathological context, focal infiltration of macrophages and lymphocytes is commonly observed. These immune cells secrete pro-fibrotic mediators—TGF-β, IL-6, and platelet-derived growth factor (PDGF)—that enhance fibroblast activation and contribute to the dysregulation of matrix metalloproteinases (MMPs).

Among the signaling pathways involved in pulmonary fibrosis, the TGF-β/Smad axis represents a central regulator of fibrogenesis. Following ligand binding, TGF-β induces the formation of a receptor complex that leads to the phosphorylation of Smad2 and Smad3. These then interact with Smad4 and translocate to the nucleus, where they regulate the transcription of profibrotic genes involved in extracellular matrix (ECM) production, including collagen type I, fibronectin, and connective tissue growth factor (CTGF).

In addition to Smad-dependent transcriptional regulation, TGF-β activates several non-canonical signaling cascades, including the MAPK, PI3K/AKT, and RhoA/ROCK pathways. These signaling branches contribute to cytoskeletal remodeling, epithelial–mesenchymal transition, and fibroblast-to-myofibroblast differentiation, thereby promoting the acquisition of a contractile, ECM-producing phenotype. Furthermore, TGF-β modulates innate and adaptive immune responses by influencing macrophage polarization toward a profibrotic phenotype, as well as regulating lymphocyte activation and cytokine secretion, thereby further amplifying the fibrotic microenvironment [14,15,16].

In parallel, the Hedgehog signaling pathway, acting primarily through Smoothened (SMO), is aberrantly reactivated in fibrotic lung tissue and contributes to disease progression. Following ligand binding (e.g., Sonic Hedgehog), inhibition of the Patched receptor is relieved, allowing SMO activation and subsequent nuclear translocation of GLI transcription fac-tors. This transcriptional program promotes fibroblast activation, myofibroblast differentiation, and extracellular matrix accumulation. Importantly, Hedgehog signaling does not act in isolation but interacts with the TGF-β pathway, creating a reinforced profibrotic circuit in which GLI-mediated transcription enhances TGF-β signaling and vice versa [17,18].

Another key signaling cascade is the Wnt/β-catenin pathway. It induces fibroblast proliferation and differentiation into myofibroblasts, increases the production of IL-1β—thereby stimulating inflammatory and pro-fibrotic responses—and promotes the synthesis of ECM [19,20,21]. The kinase TNIK, a cofactor of Wnt/β-catenin, amplifies these effects by facilitating β-catenin-mediated transcription and activating the non-canonical JNK/c-Jun pathway, which contributes to myofibroblast differentiation and ECM deposition [22]. Notably, crosstalk between Wnt/β-catenin and TGF-β/Smad signaling reinforces fibrotic responses and supports the persistence of activated myofibroblasts [23,24]. A key downstream effector of Wnt/β-catenin is Wnt1-inducible signaling pathway protein-1/CCN4 (WISP1), a secreted matricellular protein. WISP1 expression is upregulated in lungs from IPF patients and promotes fibroblast activation, differentiation into myofibroblasts, and enhanced ECM [25,26].

Several pro-inflammatory cytokines and growth factors further modulate fibrogenesis, including tumor necrosis factor-α (TNF-α) and colony stimulating factor 1 (CSF-1) [27,28,29].

S100A4 is a S100 family of small calcium-binding proteins. Under physiological conditions, S100A4 is predominantly localized intracellularly, where it contributes to cellular processes including cell motility, apoptosis, proliferation and stemness maintenance. Under pathological conditions, S100A4 is overexpressed and released into the extracellular space, where it can function as an extracellular alarmin. Extracellular S100A4 can engage pattern recognition receptors such as Toll like receptor 4 (TLR4), thereby promoting inflammatory signaling. Moreover, S100A4 can enhance canonical TGF-β signaling without interfering with baseline TGF-β activity, suggesting a mechanism through which S100A4 may amplify profibrotic signaling while preserving physiological TGF-β functions. In monocyte and whole-blood cultures, inhibition of S100A4-mediated signaling reduced the release of inflammatory cytokines, including IL-6, IL-10 and TNF-α, further supporting a role for extracellular S100A4 in linking tissue injury, inflammation and profibrotic responses [30,31].

TNF-α is a pro-inflammatory cytokine mainly produced by alveolar macrophages, epithelial cells, and activated fibroblasts. It contributes to the chronic inflammation and tissue damage that precedes fibrosis development, creating a microenvironment favorable for fibroblast activation. Furthermore, it stimulates the production of fibrogenic mediators, such as TGF-β, IL-1β, and PDGF, which enhance fibroblast-to-myofibroblast differentiation and the subsequent deposition of ECM. TNF-α induces apoptosis in alveolar epithelial cells, leading to a loss of epithelial integrity and the promotion of fibroblast proliferation and fibrotic progression [14,32].

Interleukins play a crucial role in regulating inflammatory and fibrotic responses in pulmonary fibrosis. Several members of this cytokine family modulate immune cell recruitment, fibroblast activation, and ECM remodeling. Among them, interleukin-11 (IL-11) has recently emerged as an important mediator of fibrogenesis. IL-11 is produced mainly by stromal cells and fibroblasts in response to profibrotic stimuli such as TGF-β and acts in an autocrine manner to promote fibroblast activation, myofibroblast differentiation, and ECM [33,34].

CSF-1 is a cytokine that regulates macrophage migration, proliferation, survival, and polarization. Macrophage depletion—particularly of the M2 subtype—has been shown to reduce pulmonary fibrosis. Additionally, studies have reported elevated levels of CSF-1 in bronchoalveolar lavage fluid from patients with pulmonary fibrosis, where it promotes the activation of both macrophages and fibroblasts, contributing to fibrotic processes. As a key regulator of macrophage differentiation and proliferation, inhibition of the CSF-1/CSF-1R signaling pathway disrupts macrophage production and thereby helps attenuate lung fibrosis. During the mid to late stages of radiation-induced lung injury (RILI), pro-fibrotic cytokines, such as IL-4 and IL-13, promote macrophage polarization toward the M2 phenotype. These M2 macrophages secrete and deposit ECM, leading to fibrosis and structural lung changes [35].

HMGB1 (High Mobility Group Box 1) is a nuclear protein that functions both as a transcription factor and as an extracellular alarmin, regulating inflammation and immune responses. Under conditions of cellular stress or tissue injury, HMGB1 is released into the extracellular space, where it acts as a pro-inflammatory and profibrotic signal, promoting fibroblast activation and extracellular matrix production. Preclinical and clinical studies have reported elevated HMGB1 levels in bronchoalveolar lavage fluid and serum from patients with pulmonary fibrosis, correlating with disease severity and fibrotic progression. HMGB1 also stimulates macrophage polarization toward profibrotic phenotypes, amplifying collagen and extracellular matrix deposition, contributing to lung remodeling and functional decline. Therefore, inhibition of HMGB1 signaling represents a promising therapeutic approach to simultaneously modulate inflammation and fibroblast activation, potentially reducing pulmonary fibrosis without compromising normal immune function [36,37,38].

Phosphodiesterases (PDEs), a superfamily of enzymes that hydrolyze cAMP and/or cGMP, regulate intracellular levels of these key second messengers. Among them, PDE4 is a critical component of the cAMP/protein kinase A (PKA) signaling pathway and plays a direct role in regulating cell proliferation, differentiation, and migration by modulating cAMP levels. PDE4 inhibition suppresses the release and activation of MMP-1 and MMP-9 from lung fibroblasts—key enzymes involved in ECM remodeling—and reduces the production of pro-fibrotic and pro-inflammatory cytokines such as TNF-α and TGF-β [39].

Thromboxane A_2_ receptor (TBXA2R) expression is upregulated in fibroblasts in the lungs of patients with IPF and in mouse lungs during experimental lung fibrosis. This receptor stimulates intracellular pathways such as MAPK, PI3K/AKT, and RhoA/ROCK, promoting the proliferation, migration, and differentiation of fibroblasts into myofibroblasts, thereby inducing ECM deposition. TXA_2_ can enhance TGF-β activity, promoting epithelial–mesenchymal transition. It also contributes to chronic inflammation and oxidative stress by recruiting inflammatory cells and promoting the production of reactive oxygen species (ROS) [40].

The angiotensin II type 2 receptor (AT2R) is part of the renin-angiotensin system and has a protective and anti-fibrotic role in the lung. Unlike the AT1 receptor, which mediates pro-fibrotic effects such as fibroblast proliferation, ECM deposition, and epithelial apoptosis, activation of AT2R has been associated with attenuation of fibrotic processes and improved tissue repair. AT2R expression is increased in fibroblasts from fibrotic lung tissue, and stimulation of this receptor can counteract pro-fibrotic signaling pathways, reduce collagen deposition, and promote resolution of fibrosis in preclinical models [41,42].

Finally, Lysophosphatidic Acid (LPA) signaling promotes apoptosis in lung epithelial cells while simultaneously enhancing fibroblast resistance to apoptosis. This dual effect of LPA–LPA1 signaling may help explain the paradoxical apoptotic pattern observed in IPF, characterized by increased epithelial cell death and decreased fibroblast apoptosis, both of which are thought to play key roles in the pathogenesis of fibrosis [43].

### 1.2. Therapeutic Targets and Approved Pharmacological Treatments for Pulmonary Fibrosis

Nintedanib and pirfenidone are the only pharmacological agents currently approved for the treatment of IPF. Nintedanib is an oral tyrosine kinase inhibitor (TKI) that blocks several receptors, including the receptor kinases of PDGF, fibroblast growth factor (FGF), and vascular endothelial growth factor (VEGF) [44,45,46]. Consequently, nintedanib blocks the proliferation, migration and transformation of lung fibroblasts [7]. The drug inhibits FMS-like tyrosine kinase 3 (FLT-3), RET proto-oncogene and members of the Src-family kinases, including Lck (lymphocyte-specific tyrosine-protein kinase), Lyn (tyrosine-protein kinase lyn) and Src (proto-oncogene tyrosine-protein kinase) [47,48].

Pirfenidone inhibits the production of TNF-α, TGF-β, collagen, and fibroblast proliferation [49]. The main adverse events for the two drugs are mostly gastrointestinal and include nausea, diarrhea and loss of appetite. Due to adverse events, the dosage may be reduced or treatment temporarily discontinued [50].

These antifibrotic agents slow the progression of lung function decline, but their effectiveness is limited: the disease has irreversible outcomes that lead to respiratory failure, with progressive impairment of gas exchange at the pulmonary level [51,52].

Therefore, the limited efficacy of current antifibrotic treatments highlights the need for the development of novel drugs that can interrupt disease progression and potentially cure this condition.

## 2. Materials and Methods

### 2.1. Study Design and Search Strategy

This scoping review was conducted in accordance with the Preferred Reporting Items for Systematic Reviews and Meta-Analyses extension for Scoping Reviews (PRISMA-ScR) guidelines [53], ensuring both transparency and reproducibility. Medical Subject Headings (MeSH) terms and Boolean operators were used, based on existing literature, as follows: “(Pulmonary fibrosis)” AND “(Drug Therapy)”. The literature search was conducted only on studies registered on ClinicalTrials.gov (U.S. National Library of Medicine, Bethesda, MD, USA; https://clinicaltrials.gov). The last literature search was conducted on March 2026.

The search strategy was conducted through the selection of the following filters:

Condition or Disease: “pulmonary fibrosis”, “Drug Therapy”;

Other terms: “treatment” or “novel therapy” or “drug, treatment, therapy” or “drug, new treatment, novel therapy”;

Sex: all;

Age: adult and older adult;

Study phase: Phase II or III;

Study type: interventional;

Study results: studies with and without results;

Date range: from 1 January 2014 to 31 December 2025;

Recruitment status: recruiting (currently recruiting participants), enrolling by invitation (participants are selected from a population or group of people decided on by the researchers in advance), active not recruiting (the study is ongoing, and participants are receiving an intervention or being recruited or enrolled), active (the study is ongoing, but potential participants are not currently being recruited or enrolled), not yet recruiting (the study has not started recruiting participants), terminated (terminated early without subsequent restart), or completed (completed with no additional examinations or treatments on the enrolled patients).

### 2.2. Literature Search Results

The initial search identified 208 records. After removing 27 duplicates, 181 studies were screened by title and abstract, resulting in the exclusion of 133 records. Fourty-eight records were assessed for eligibility, and after a full text examination 19 studies met the inclusion criteria.

### 2.3. Inclusion and Exclusion Criteria

The review was preregistered on the Open Science Framework prior to data collection (DOI 10.17605/OSF.IO/VEJG4).

The inclusion and exclusion criteria were defined using the PCC framework (Population, Concept, Context):

P: patients with IPF;

C: novel pipeline treatments in Phase II and Phase III clinical trials;

C: clinical trial settings involving adults with IPF.

The PCC question was: “Which novel pharmacological agents are currently in clinical trials for pulmonary fibrosis, and what are the mechanisms of action and emerging therapeutic targets being studied?”

The inclusion criteria for the studies were as follows: adults and older adults; Phase II and Phase III clinical trials; range of publication dates 1 January 2014 to 31 December 2025; randomized and non-randomized studies; active, terminated or completed studies with no published results or without results; English language; and full text available.

The exclusion criteria were: pediatric patients, drugs and formulations already on the market for this indication, Phase I clinical trials completed or terminated before 2025, studies with results already published, studies focused on a different disease, studies investigating molecules whose mechanism of action has not yet been fully elucidated, letters, protocols, reviews (clinical review, systematic review), meta-analysis, and full text not available. Trials with available results were excluded to maintain focus on the current investigational pipeline.

### 2.4. Study Selection, Data Charting, and Analysis

**Figure 1:**
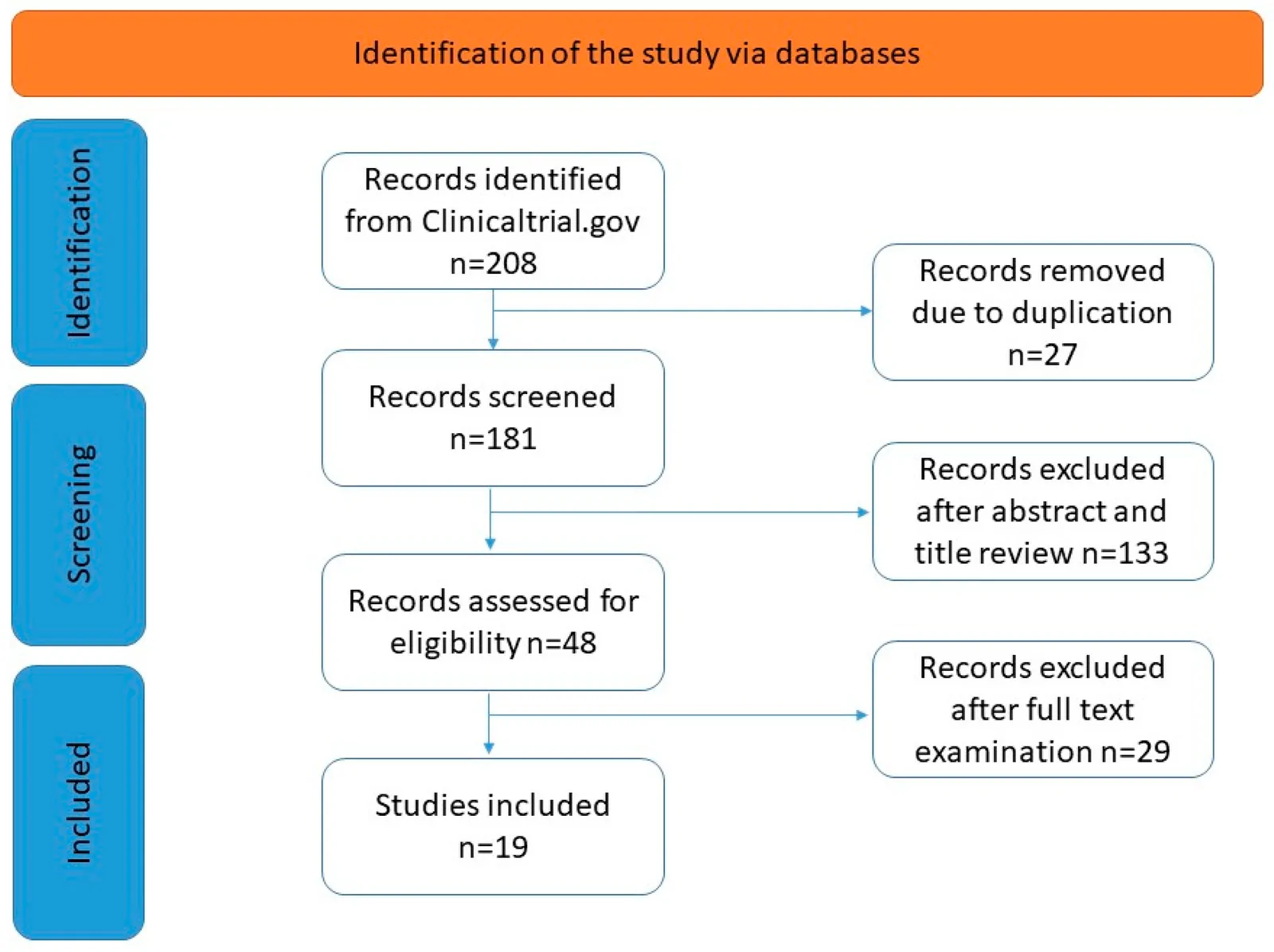
PRISMA 2020 flow chart of the studies included in the scoping review.

Study selection followed the PRISMA flow diagram (Figure 1). Duplicates were removed using the Rayyan tool. Then all articles were screened by title and abstract to eliminate the studies that did not meet the inclusion criteria. A full-text examination was used to assess eligibility for inclusion. Two reviewers (M.C. and M.E.N.) screened and assessed the articles independently, resolving any issues through discussion, while a third author (A.P.) reviewed the overall process.

## 3. Results

Nineteen studies were included and the main characteristics are summarized in Table 1. Twelve studies are in recruiting status [54,55,56,57,58,59,60,61,62,63,64,65], two in active not recruiting status [66,67], four in not yet recruiting status [68,69,70,71] and one completed without results [72]. In addition, fifteen are in phase II [56,57,58,59,60,61,62,63,64,65,67,68,69,70,72], three in phase III [55,66,71] and one in phase II/III [54]. The studies analyzed predominantly employed randomized allocation, with the majority utilizing a parallel assignment interventional model [54,56,57,58,66,68,72]. The masking strategy most commonly used is quadruple blinding, involving participants, care providers, investigators, and outcomes assessors [54,57,58,66,68]. A smaller number of trials used triple masking [55,64], generally excluding the care provider or the outcomes assessor. Double masking, involving only participants and investigators, was reported in three studies [57,68,71].

In addition, two extension trials adopted an open-label design without masking and with single-group assignment, primarily aimed at evaluating long-term safety and tolerability [57,67] (Table 1).

### 3.1. Sufenidone

Sufenidone (SC1011) is a new pyridone derivative designed to improve the antifibrotic activity of pirfenidone [13]. In preclinical studies, it has demonstrated antifibrotic and anti-inflammatory effects, partly through inhibition of the TGF-β1 signaling pathway, a key mechanism involved in fibrosis and also associated with the antifibrotic activity of pirfenidone.

In the lung, TGF-β1 is produced by various cell types in response to epithelial injury caused by environmental or other factors. These include alveolar epithelial cells, endothelial cells, fibroblasts, myofibroblasts, and inflammatory cells such as macrophages and neutrophils. There is evidence that sufenidone, like pirfenidone, can modulate the production of TNF-α, IL-1β, IL-6, IL-8, IL-10, and IFN-γ, as well as the expression of adhesion molecules and factors involved in matrix remodeling [73,74].

Two Phase I studies evaluated the safety, tolerability, and pharmacokinetic profile of the immediate-release and modified-release formulations of SC1011 in healthy subjects. Overall, the studies showed that sufenidone was generally safe and well tolerated, with mostly mild to moderate adverse events, primarily gastrointestinal symptoms such as nausea and vomiting [13].

The ongoing Phase II/III study of SC1011 (NCT06125327) is a randomized, double-blind study. A total of 210 participants will be divided into three groups—two receiving active treatment and one receiving placebo—with an interim analysis at 26 weeks to select the optimal dose for the subsequent 52-week treatment period, followed by a 4-week safety monitoring phase. The primary outcome will be to evaluate the drug’s efficacy and safety in patients with IPF by calculating the forced vital capacity (FVC) [54].

### 3.2. HEC585 (Yinfenidone)

HEC585 is a pyrimidone analog derived from pirfenidone with preclinically improved metabolic stability, longer half-life and higher activity [75]. Similarly to pirfenidone, it appears to inhibit the activity of transforming growth factors, including TGF-α and TGF-β.

HEC585 was first evaluated in phase I studies in healthy volunteers to assess pharmacokinetics, safety, and tolerability. This was followed by a recruiting phase IIb trial in patients with progressive fibrosing interstitial lung disease (NCT05139719), which included a broader population encompassing IPF and other fibrosing ILDs. In this study, the efficacy and safety of two doses of HEC585 tablets will be compared with placebo in 110 patients. The primary endpoint was change from baseline in FVC at 24 weeks [73].

In a phase III trial that is not currently recruiting (NCT07082842), the efficacy and safety of HEC585 will be evaluated in patients with IPF, compared with placebo and pirfenidone. In total, 472 patients will be enrolled and divided into three treatment groups: HEC585 tablets (once daily), placebo, and pirfenidone. The primary outcome will be the change in FVC from baseline at 52 weeks [71].

### 3.3. BI765423

BI765423 is a human monoclonal antibody that blocks IL11, reducing fibroblast activation and their differentiation into myofibroblasts. This ultimately leads to decreased production and deposition of ECM.

The safety, tolerability, pharmacokinetics, pharmacodynamics, and efficacy of BI 76542 in patients with IPF will be evaluated every 4 weeks in a randomized, phase II clinical trial: NCT07036523. The study will compare the drug with placebo to evaluate differences in lung function after 3 months of treatment, as well as changes in biomarkers associated with lung health. A total of 71 participants will be randomly assigned to receive either placebo or BI765423 as an infusion every four weeks, during which they may continue their regular treatment for IPF [62].

### 3.4. TDI01

TDI01 suspension selectively inhibits downstream ROCK2, an enzyme essential for cell movement and cytoskeleton formation. Normally, ROCK2 mediates inflammation and vascular permeability, which are critical in lung injury. Treating mice with TDI01 resulted in a reduction in the inflammatory response, a reduction in pulmonary edema, and improved vascular function. TDI01 also inhibits adhesion molecules (ICAM1, VCAM1) at the endothelial cell level, which makes the endothelium more permeable and contributes to its dysfunction [76].

TDI01 is currently being evaluated in a Phase II trial assessing the efficacy and safety of different doses compared with control in patients with IPF (NCT06102083). The study includes a 4-week screening period, a 24-week treatment period, a 28-week extension period, and a 2-week safety follow-up period, during which a total of 120 patients will be enrolled. All subjects will be randomized in a 1:1:1 ratio to receive 24 weeks of treatment. At Week 24, subjects will be assessed for the primary efficacy endpoints and will subsequently enter the extension period [68].

### 3.5. INS018_055 Rentosertib

INS018_055 blocks TNIK, thereby preventing the fibrotic activity induced by pro-fibrotic mediators.

The molecule was designed using artificial intelligence, an innovative approach to the development of targeted therapies. Once the structure of the TNIK hinge region was elucidated, the AI system generated molecules capable of forming hydrogen bonds with the Cys108-NH of the hinge region. In addition, a hydrophobic moiety was incorporated into the molecule to occupy the deep binding pocket [77].

A completed Phase IIa study (NCT05938920) conducted in China evaluated the safety and tolerability of INS018_055 orally administered for up to 12 weeks in 71 adult subjects with IPF compared to placebo. Patients were randomly assigned in a 1:1:1:1 ratio to receive drug (at a dose of 30 mg (QD), 30 mg (BID) or 60 mg (QD)) or placebo (QD) for 12 weeks, along with the continued use of standard of care medications. The drug demonstrated safety and tolerability. Rare adverse events were related to hepatic toxicity, diarrhea and hypokalemia [78].

NCT05975983, a Phase IIa study, will be conducted in the USA to evaluate the safety and tolerability of INS018_055 orally administered for up to 12 weeks in 60 adult subjects with IPF compared to placebo. The study will include multiple dose levels (e.g., 30 mg QD, 30 mg BID, 60 mg QD) and will allow subjects to continue standard-of-care antifibrotic therapy (such as nintedanib or pirfenidone) if already prescribed. Participants will undergo regular safety assessments and pulmonary function testing throughout the study period [56].

### 3.6. Axatilimab

Axatilimab, a humanized IgG4 monoclonal antibody targeting CSF-1R—a cell surface protein implicated in the development of fibrotic diseases—was approved in August 2024 for the treatment of chronic graft-versus-host disease (cGVHD) [79]. An ongoing Phase II study (NCT06132256) is evaluating the effects of intravenous administration of axatilimab in patients with IPF. This randomized, double-blind, placebo-controlled, multicenter study will enroll approximately 135 participants who will receive either axatilimab or placebo in a 2:1 ratio. The trial will last 26 weeks, during which lung function parameters, including FVC and DLCO, general health status, and quality of life will be assessed through standardized questionnaires, while safety and tolerability will be closely monitored with laboratory tests, vital signs, and treatment emergent adverse events [58].

### 3.7. BI 1819479

The mechanism of action of BI 1819479 in IPF appears to be based on inhibition of the enzyme autotaxin, leading to reduced production of LPA. This, in turn, decreases stimulation of LPA receptors involved in fibroblast/myofibroblast activation and ECM deposition in IPF [80].

NCT06335303 is a randomized, double-blind, Phase II study testing three different doses of BI 1819479 in patients receiving or not receiving standard-of-care therapy. Participants will be treated for at least one year, during which lung function tests will be performed, and potential side effects will be assessed. The primary objective is to evaluate the efficacy, safety, and tolerability of the drug [72].

### 3.8. HRS-9813

HRS-9813 is another inhibitor of ATX. NCT07192939 is a recruiting, multicenter, double-blind Phase II trial designed to evaluate the efficacy and safety of HRS-9813 in subjects with Progressive Pulmonary Fibrosis (PPF) and IPF. A total of 270 patients will be randomly divided into three groups depending on the intervention: high-dose HRS-9813 capsules, low-dose HRS-9813 capsules, and placebo capsules. The primary outcome is FVC. The baseline period lasts until 26 weeks after administration [59].

### 3.9. MTX-463

MTX-463 is an Human IgG1 monoclonal antibody acting on WISP1, an effector of Wnt/β-catenin that promotes fibroblast activation, differentiation into myofibroblasts, and extracellular matrix deposition. In a phase IIa recruiting trial, 164 participants with IPF will receive an IV infusion every 4 weeks while continuing standard-of-care therapies. The primary outcome is the change in FVC at 24 weeks, with secondary endpoints including safety, tolerability, and biomarkers of fibrosis [61].

### 3.10. BMS-986278 Admilparant

Admilparant is a second-generation LPA antagonist that was developed to avoid the serious hepatic side effects associated with the first-generation BMS-986020 [81].

In a Phase I study (NCT03429933), admilparant reduced blood pressure without affecting heart rate, so in the Phase II study (NCT04308681), participants who had a reduction in blood pressure were treated with 10 mg instead of 30 or 60 mg. The main adverse event was diarrhea; others were reduced blood pressure, cough, dyspnea and nausea. The trial concluded that admilparant improves lung function and has a good safety profile, thus supporting its evaluation in Phase III studies [82].

Admilparant is currently undergoing a multicenter, randomized, double-blind, placebo-controlled Phase III study (NCT06003426). The aim is to evaluate the efficacy, safety, and tolerability of BMS-986278 in individuals diagnosed with IPF under standard-of-care treatment. Participants receiving pirfenidone or nintedanib must have been on a stable dose for at least 90 days prior to screening, while participants not receiving pirfenidone or nintedanib must not have received either medication within 28 days prior to screening [66].

### 3.11. SB17170

SB17170 is an inhibitor of HMGB1, a key damage-associated molecular pattern that drives fibroblast activation and extracellular matrix deposition in idiopathic pulmonary fibrosis. By blocking HMGB1 signaling, SB17170 may reduce inflammation-driven fibrosis and slow disease progression.

NCT06747923 is a recruiting phase II trial in which the change in FVC will be compared to placebo by administering SB17170 to moderate to severe patients with IPF. Thirty subjects will be randomized into test group 1, test group 2 and a control group (Placebo for SB17170) in 2:2:1 and take two capsules once daily for 12 weeks. Safety and tolerability at 12 weeks after randomization and efficacy at 4 and 12 weeks will be assessed [60].

### 3.12. Ifetroban

Ifetroban is a selective antagonist of TBXA2R, which is expressed in various cell types, including fibroblasts [83].The drug is currently under investigation in a Phase II, randomized, double-blind, placebo-controlled study (NCT05571059). The aim of the trial is to determine the safety and efficacy of the drug in 128 patients with IPF, treated once daily for twelve months, during which they will be monitored. Efficacy will be measured through pulmonary function parameters (such as FVC) and other biomarkers, including data derived from CT imaging analyses [57].

### 3.13. Buloxibutid

Buloxibutid is described as an oral AT2R agonist, targeting alveolar epithelial type 2 cells to drive epithelial repair and reduce pro-fibrotic signaling in idiopathic pulmonary fibrosis.

In NCT06588686, a recruiting, randomized, double-blind, placebo-controlled, multicenter trial, will evaluate the efficacy and safety of two doses of Buloxibutid over 52 weeks in subjects with IPF. The trial is planned to enroll 360 participants who are on stable licensed IPF therapy or who are currently not treated with a licensed IPF therapy. Of these, a portion will receive on oral buloxibutid 100 mg BID, a portion participants on oral buloxibutid 50 mg BID, and a portion on oral placebo BID for 52 weeks. The primary measurement will be based on spirometry, measuring the FVC [63].

### 3.14. BI 1015550 Nerandomilast

Nerandomilast is more selective for the PDE4B subtype compared with other drugs in the same class, such as roflumilast and apremilast, which inhibit PDE4D and cause adverse gastrointestinal reactions, like nausea, vomiting, and diarrhea. Nerandomilast is a strong inhibitor of TNF-α and IL-2, and since these factors are involved in the inflammatory phase of pulmonary fibrosis, it may have anti-inflammatory and immunomodulatory effects [84].

In a Phase I trial (NCT03230487), enrolled participants received nerandomilast in escalating doses of 36 and 48 mg, multiple escalating doses of 6 and 12 mg, or placebo twice daily for 14 days. Two different studies were conducted: in the single ascending dose (SAD) group, only the patients were blinded, and they fasted before administration of the drug, while in the multiple ascending dose (MAD) group, both the patients and the researchers were blinded, and the drug was administered in a fed state. The aim of this trial was to analyze adverse events and the tolerability of the drug. Adverse events were: nervous system disorders (headache) and gastrointestinal disorders (diarrhea, nausea, abdominal pain and constipation). No deaths or serious adverse events were reported. Laboratory tests revealed an increase in triglycerides in one participant, which was suspected to be drug-related [85].

A phase II study (NCT04419506) evaluated the effects of the drug in patients with IPF, whether or not they were receiving standard-of-care therapy. This trial showed that participants receiving treatment had better lung function, regardless of the antifibrotic drugs they were taking (nintedanib and pirfenidone), compared with those receiving a placebo.

A Phase III trial (NCT06238622) (FIBRONEER™-ON) is a study that will enroll only patients who have previously been treated with the drug, including individuals with IPF and PPF. The purpose of the study is to understand how well participants tolerate long-term treatment with nerandomilast. Researchers will also examine whether the medicine helps improve lung function and delays worsening symptoms, hospital visits, or death. Participants will take nerandomilast tablets for up to 1 year and 10 months and may continue their usual treatments for pulmonary fibrosis during the study. Throughout the study, participants will have regular appointments with their doctors. During these visits, doctors will monitor any health issues and conduct routine lung function tests [55].

### 3.15. HSK4459

Similarly to nerandomilast, HSK44459 is a selective PDE4B inhibitor developed to provide anti-inflammatory and antifibrotic effects with fewer adverse effects than non-selective PDE4 inhibitors [39]. NCT06764862 is a randomized, double-blind, placebo-controlled, parallel-group phase II clinical trial evaluating the efficacy and safety of HSK44459 tablets in patients with idiopathic pulmonary fibrosis (IPF). In this not-yet-recruiting study, the change from baseline in FVC at week 12 will be assessed in 120 patients. Participants will be randomized into three treatment groups: HSK44459 dose 1, HSK44459 dose 2, and placebo. The drug will be administered orally twice daily for 12 weeks [69].Another phase II trial that is not yet recruiting, NCT07019090, will enroll approximately 120 patients with IPF who have previously completed treatment in earlier clinical trials. The aim will be to evaluate the long-term efficacy and safety of HSK4459 [70].

### 3.16. ENV-101 (Taladegib)

ENV-101 is an inhibitor of the Hedgehog signaling pathway. By blocking SMO, it reduces fibroblast activation and myofibroblast differentiation, thereby limiting extracellular matrix deposition and the progression of fibrosis in IPF [86]. In a completed multicenter, randomized, double-blind, placebo-controlled trial (NCT04968574), the tolerability of the drug was evaluated in 41 patients with IPF. Treatment for 12 weeks resulted in an improvement in percent predicted FVC and reductions in imaging markers of fibrosis compared with placebo, with an acceptable safety profile. These findings supported further investigation in a larger phase 2b trial: NCT06422884. This 6-month, multicenter, randomized, double-blind, dose-ranging trial will investigate the efficacy, antifibrotic activity, and safety of ENV-101 in adults with IPF. Participants are assigned to one of three ENV-101 dose levels or placebo, with continuation of standard-of-care therapies (nintedanib or pirfenidone). The trial aims to evaluate the impact of ENV-101 on lung function and key fibrosis biomarkers and to identify the optimal dosing regimen for subsequent Phase 3 studies [67].

### 3.17. CAL 101 (Idelalisib)

CAL101 is a humanized IgG4 monoclonal antibody that binds S100A4, a calcium-binding protein of the S100 family that acts as an extracellular alarmin. By neutralizing extracellular S100A4, CAL101 inhibits S100A4-mediated TLR4 activation and the enhancement of canonical TGF-β signaling while preserving baseline TGF-β activity and reduces the release of S100A4-driven inflammatory cytokines, thereby exerting combined anti-inflammatory and antifibrotic effects [87]. Its potential use in IPF is currently being evaluated: NCT06736990. In this recruiting phase II trial, 150 participants with IPF will be enrolled to compare CAL101 with placebo with regard to the change from baseline in FVC. The study includes a 28-day screening period and a 16-week follow-up period, during which IV infusions of CAL101 or placebo will be administered once every 4 weeks for a total of 24 weeks [64].

## 4. Discussion

**Figure 2:**
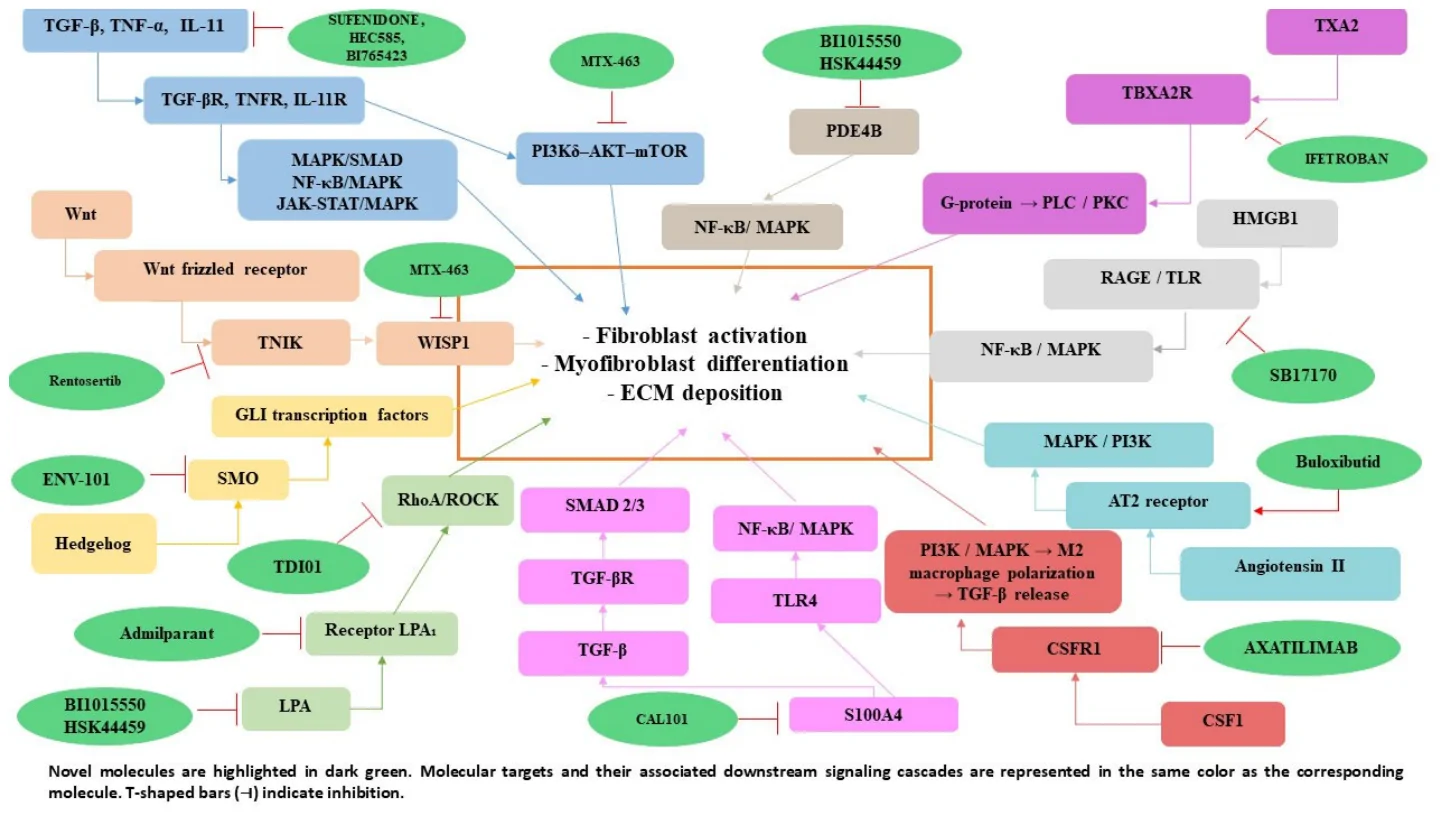
Mechanisms involved in disease progression and the potential points of therapeutic intervention.

**Figure 3:**
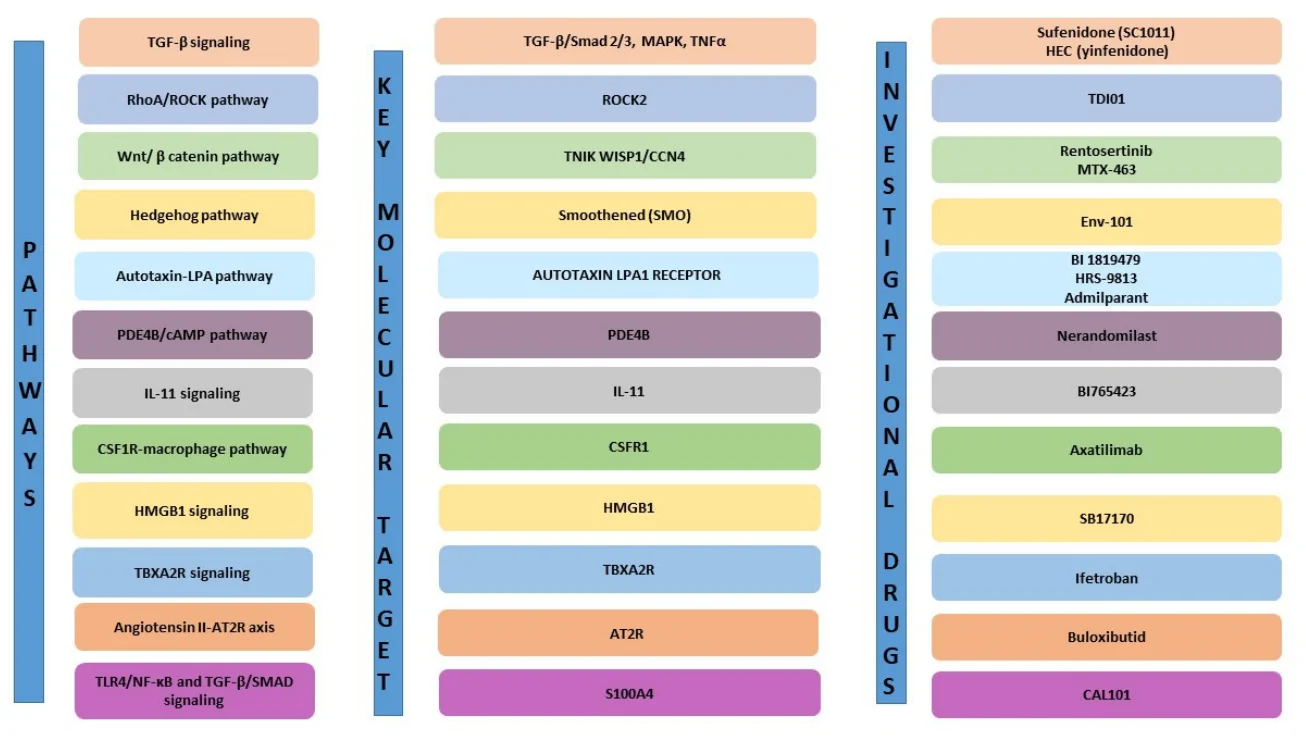
Pathogenic pathways in IPF and corresponding investigational drugs.

This scoping review describes new therapeutic approaches for the treatment of IPF currently undergoing Phase II–III RCTs. In the 19 studies analyzed, the total number of participants was 6508. The clinical studies investigate key signaling pathways and molecular targets, including MAPK, RhoA/ROCK, PDE4B/cAMP, Wnt/β-catenin, Hedgehog/SMO, IL-11/STAT3, and LPA/autotaxin and Angiotensin II Type 2, as well as extracellular receptors and mediators such as CSF1R, TBXA2R and WISP1 (Table 2) (Figure 2 and Figure 3).

The analysis focuses on the drug development pipeline with the aim of highlighting emerging therapies beyond standard treatments and outlining potential future directions in IPF management.

## 5. Translational Challenges in IPF Therapeutic Development

### 5.1. Biological Complexity of Profibrotic Signaling in IPF

IPF represents a complex therapeutic challenge, as fibrotic processes are regulated by interconnected signaling networks characterized by redundancy and integration with essential physiological functions [88,89]. This complexity is further amplified by important crosstalk among profibrotic pathways, including TGF-β, Wnt/β-catenin, Hedgehog, and RhoA/ROCK, which act in a coordinated manner to sustain fibroblast activation and ECM remodeling.

Consequently, inhibition of a single molecular target is often insufficient, since compensatory mechanisms and alternative signaling routes can partially replace its function, thereby reducing the overall impact of targeted therapies. In addition, many mediators involved in fibrogenesis also play essential roles in physiological processes such as tissue repair, immune regulation, and maintenance of epithelial integrity. This functional overlap limits therapeutic selectivity and contributes to a relatively narrow therapeutic window for several pharmacological targets.

The clinical presentation of IPF adds another layer of complexity: patients often exhibit variability in disease progression, suggesting that fibrotic activity may be driven by different dominant mechanisms depending on the individual or stage of disease. This variability likely contributes to the inconsistent treatment responses observed in clinical studies.

### 5.2. Lessons Learned from Clinical Development and Future Directions

Among the interconnected profibrotic pathways involved in IPF, TGF-β signaling represents the most established reference model for fibrogenesis and is supported by robust preclinical evidence. However, its clinical efficacy remains limited, likely due to its pleiotropic involvement in essential physiological processes, including tissue homeostasis and immune regulation, which narrows its therapeutic window and increases susceptibility to dose-limiting adverse effects. In addition, the complexity of its activation within the tissue microenvironment, together with the presence of redundant profibrotic networks, may reduce the efficacy of isolated targeting strategies, thereby contributing to both the persistence of fibrosis despite pathway inhibition and the occurrence of systemic adverse events following pathway modulation [15,88,89].

Clinical evidence from approved antifibrotic therapies such as pirfenidone suggests a limited disease-modifying effect, mainly reflected by a partial attenuation of disease progression rather than complete disease control [50]. Furthermore, frequent dose reductions or treatment discontinuations due to adverse events may further limit drug exposure and consequently reduce overall therapeutic benefit. Similar considerations may also apply to investigational agents targeting the same axis, including sufenidone and HEC585, which may share class-related limitations associated with TGF-β modulation, further supporting the concept that inhibition of this pathway alone may be insufficient to achieve robust clinical efficacy [5].

Comparable limitations have also been observed with other profibrotic pathways, including LPA/autotaxin, Wnt/β-catenin, Hedgehog/SMO, RhoA/ROCK, and PDE4 signaling. Despite improvements in pharmacological optimization and target selectivity, these systems remain involved in fundamental physiological functions such as vascular regulation, cellular motility, tissue repair, development, and modulation of inflammatory responses. Their broad biological involvement may therefore contribute to a narrow therapeutic window and increase the risk of dose-limiting toxicities. Moreover, the extensive crosstalk among profibrotic networks promotes compensatory signaling mechanisms that may attenuate the clinical impact of single-pathway inhibition. In this scenario, the negative or inconclusive results reported with agents such as ziritaxestat or BMS-986020 may reflect not only pharmacological limitations, but also intrinsic biological constraints associated with the complexity and redundancy of fibrotic signaling [90,91]. These findings suggest that clinical failure in IPF may not necessarily reflect inadequate target selection or insufficient pharmacological potency, but rather the intrinsic limitations of attempting to modulate isolated pathways within a highly interconnected fibrotic network.

In this context, new therapeutic targets are emerging, including CSF1R, HMGB1, WISP1, IL-11, S100A4, the thromboxane receptor, and the AT2R axis. Compared with classical profibrotic pathways, these targets may offer more selective opportunities to modulate specific components of the fibrotic microenvironment, including fibroblast activation, recruitment and polarization of profibrotic macrophages, inflammatory signaling, and extracellular matrix deposition. S100A4 represents a particularly interesting emerging target because it acts as an alarmin that links tissue injury to inflammatory and profibrotic signaling. Extracellular S100A4 can activate TLR4-dependent inflammatory pathways and enhance canonical TGF-β signaling, thereby providing a mechanistic link between innate immune activation and fibrotic remodeling. Targeting extracellular S100A4 may therefore offer the possibility of modulating pathological inflammatory and profibrotic signaling while preserving the physiological intracellular functions of S100A4 and the basal activity of TGF-β [25,26,30,31,36,40,41,43]. 

The variability of outcomes observed with antifibrotic strategies, including both approved therapies and compounds currently under clinical development, may not solely reflect intrinsic limitations of individual molecules, but also methodological aspects related to clinical trial design. Efficacy is frequently assessed using short-term surrogate endpoints, which may not reliably capture true disease-modifying effects in the long term. Moreover, the limited molecular stratification of patients enrolled in clinical studies often includes clinically heterogeneous populations treated as biologically uniform, which may obscure treatment effects restricted to specific molecular or phenotypic subgroups and thereby contribute to variability or lack of statistical significance across trials. Furthermore, variability in background therapies may further confound the interpretation of both investigational and approved interventions, limiting the ability to isolate treatment-specific effects.

Overall, the success of future therapies for IPF will depend not only on the biological validity of individual molecular targets, but also on their ability to function within a complex and dynamic disease system. It is likely that the greatest clinical benefit will derive from combination strategies and precision medicine approaches, rather than from the replacement of current standard-of-care therapies with single novel agents. Given the extensive crosstalk among profibrotic pathways, combining agents targeting complementary mechanisms may enhance therapeutic efficacy and help overcome compensatory signaling networks. Furthermore, improved patient stratification through molecular profiling and biomarker-guided approaches may facilitate the identification of individuals most likely to benefit from specific targeted therapies. In this context, at present, lung transplantation may represent the only viable alternative in patients with advanced disease, while emerging therapies are expected to play a complementary role, contributing to the slowing of disease progression, delaying the need for transplantation, and stabilizing clinical decline.

## 6. Strengths and Limitations

This scoping review has several methodological strengths. Firstly, it was conducted in accordance with PRISMA-ScR guidelines and preregistered on the Open Science Framework, ensuring transparency, reproducibility, and traceability. In addition, two reviewers independently selected the studies and a third author verified the selection, minimizing selection bias. The use of the PCC framework allowed a clear definition of the research question and facilitated systematic mapping of the drug development pipeline in IPF. These features provide an up-to-date, methodologically robust overview of ongoing Phase II–III clinical trials in IPF.

However, several limitations should be acknowledged. The included studies exhibit heterogeneity due to differences in clinical development phases, the inclusion of an open-label extension, and variability in enrolled populations, some of which included patients with PPF or SSc-ILD. The investigational drugs target diverse molecular pathways, limiting direct comparisons and precluding conclusions about the robustness of individual targets. Most primary endpoints are short-term surrogate measures, which have limited sensitivity and specificity for true disease progression, preventing assessment of long-term efficacy or safety. Additionally, reliance on ClinicalTrials.gov introduces constraints such as delayed updates, undocumented protocol modifications, and incomplete methodological details. Consistent with the nature of scoping reviews, no formal risk-of-bias assessment or qualitative appraisal of the studies was performed. A systematic review or meta-analysis could not be performed because the included RCTs are still ongoing and have not yet published their results. Consequently, no efficacy or safety data were available for quantitative synthesis. Furthermore, this methodological choice limits the interpretation of the actual therapeutic efficacy and long-term clinical impact of the investigational agents, as conclusions can currently be drawn only from their biological rationale, mechanisms of action, and trial design characteristics. These limitations are intrinsic to the scoping review methodology and reflect the exploratory and evolving nature of the current IPF drug development landscape, rather than deficiencies in study design or conduct.

## 7. Conclusions

This scoping review highlights the expansion of investigational therapies for IPF, reflecting a gradual shift from broad antifibrotic approaches toward increasingly specific molecular targeting strategies. Despite these advances, current evidence suggests that clinical translation remains limited, and the long-term clinical benefit of investigational drugs has yet to be established. At present, none of the investigational therapies appear capable of replacing existing antifibrotic treatments or lung transplantation in patients with advanced disease.

The failure of multiple pathway-specific strategies suggests that IPF pathogenesis cannot be effectively addressed through isolated molecular targeting alone. Rather, biological redundancy, pathway compensation, and the physiological relevance of the involved signaling networks represent fundamental barriers to therapeutic success.

Future progress in IPF therapy will likely require a shift toward integrated strategies, including combination regimens, biomarker-driven patient stratification, and adaptive clinical trial designs. A deeper understanding of disease heterogeneity and pathway interplay will be essential to translate molecular advances into meaningful clinical benefit.

## Figures and Tables

**Table 1:** Main characteristics of the selected studies.

Official Title/NCT	Study Phase	Enrollment	Study Design	Status	Primary Outcome	Drug Under Investigation
A Randomized, Double-blind, Placebo-controlled Phase II/III Trial Evaluating the Efficacy and Safety of Sufenidone (SC1011) Tablets in Patients With Idiopathic Pulmonary Fibrosis (IPF). NCT06125327 [54]	II/III	210	-Allocation: Randomized; -Interventional Model: Parallel Assignment; -Masking: Quadruple (Participant, Care Provider, Investigator, Outcomes Assessor)	Recruiting	Annual Rate of Decline in FVC Over 52 Weeks	Sufenidone (SC1011)
A Phase II, Multi-center, Randomized, Double-blinded, Placebo-controlled Clinical Study Evaluating the Efficacy and Safety of TDI01 Suspension for the Treatment of Idiopathic Pulmonary Fibrosis (IPF). NCT06102083 [68]	II	120	-Allocation: Randomized; -Interventional Model: Parallel Assignment; -Masking: Quadruple (Participant, Care Provider, Investigator, Outcomes Assessor)	Not yet recruiting	Change From Baseline in FVC (mL) at Week 24	TDI01
An Open-label Extension Trial of the Long-term Safety and Efficacy of BI 1015550 Taken Orally in Patients With Idiopathic Pulmonary Fibrosis (IPF) and Progressive Pulmonary Fibrosis (PPF) (FIBRONEER™-ON). NCT06238622 [55]	III	1700	-Allocation: N/A; -Interventional Model: Single Group; -Assignment -Masking: None (Open Label)	Recruiting	Occurrence of any adverse event over the course of the extension trial (yes/no), i.e., up until the follow-up/end of study visit planned at the latest at week 99	Nerandomlast
A Randomised, Double-blind, Placebo-controlled, Dose-finding Study Evaluating Efficacy, Safety, and Tolerability of Different Oral Doses of BI 1819479 Over at Least 24 Weeks in Patients With Idiopathic Pulmonary Fibrosis (IPF) NCT06335303 [72]	II	320	-Allocation: Randomized; -Interventional Model: Parallel Assignment; -Masking Triple (Participant, Care Provider, Investigator)	Completed	Annual rate of decline in FVC	BI 1819479
A Phase IIa, Randomized, Double-Blind, Placebo-Controlled Study Evaluating the Safety, Tolerability, Pharmacokinetics, and Efficacy of INS018_055 Administered Orally to Subjects With Idiopathic Pulmonary Fibrosis (IPF). NCT05975983 [56]	II	60	-Allocation: Randomized; -Interventional Model: Parallel Assignment; -Masking Double (Participant, Investigator)	Recruiting	Percentage of patients who have at least 1 treatment emergent adverse event (TEAE)	Rentosertib
A Randomized, Double-Blind, Placebo-Controlled Phase II Study to Determine the Safety and Efficacy of Oral Ifetroban in Patients With Idiopathic Pulmonary Fibrosis (IPF). NCT05571059 [57]	II	128	-Allocation: Randomized; -Interventional Model: Parallel Assignment; Masking: Quadruple (Participant, Care Provider, Investigator, Outcomes Assessor)	Recruiting	Annual rate of decline in FVC in mL	Ifetroban
A 26-Week, Randomized, Double-Blind, Placebo-Controlled, Multi-center Study to Evaluate the Efficacy, Safety, and Tolerability of Axatilimab in Subjects With Idiopathic Pulmonary Fibrosis (IPF). NCT06132256 [58]	II	135	-Allocation: Randomized; -Interventional Model: Parallel Assignment; -Masking Quadruple (Participant, Care Provider, Investigator, Outcomes Assessor)	Recruiting	Annualized rate of decline in morning pre-dose trough FVC	Axatilimab
A Multicenter, Randomized, Double-blind, Placebo-controlled, Phase 3 Study to Evaluate the Efficacy, Safety, and Tolerability of BMS-986278 in Participants With Idiopathic Pulmonary Fibrosis. NCT06003426 [66]	III	1185	-Allocation: Randomized; -Interventional Model: Parallel Assignment; -Masking Quadruple (Participant, Care Provider, Investigator, Outcomes Assessor)	Active not recruiting	-Number of participants that experience spontaneous syncopal events -Absolute change from baseline in FVC measured in mL	Admilparant
Multicenter, Randomized, Double-blind, Placebo-controlled Phase II Trial to Evaluate the Efficacy and Safety of HRS-9813 in Subjects With Pulmonary Fibrosis. NCT07192939 [59]	II	270	-Allocation: Randomized; -Interventional Model: Parallel Assignment; -Masking Quadruple (Participant, Care Provider, Investigator, Outcomes Assessor)	Recruiting	FVC as a percentage of the predicted value	HRS-9813
A Phase 2 Trial of ENV-101 in Patients With Lung Fibrosis (WHISTLE-PF Trial). NCT06422884 [67]	II	213	-Allocation: Randomized -Interventional Model: Parallel Assignment -Masking: Triple (Participant, Investigator, Outcomes Assessor)	Active, not recruiting	Change in percent predicted forced vital capacity (ppFVC) compared to placebo	ENV-101 (taladegib)
Confirmatory Clinical Study of HEC585 Tablets in Patients With IPF. NCT07082842 [71]	III	472	-Allocation: Randomized -Interventional Model: Parallel Assignment -Masking: Quadruple (Participant, Care Provider, Investigator, Outcomes Assessor)	Not yet recruiting	Change in FVC from baseline at 52 weeks	HEC585 (yinfenidone)
A Study to Explore the Therapeutic Effect of HEC585 on Delaying Forced Vital Capacity (FVC) Decline and Tolerance in Progressive Fibrosing Interstitial Lung Disease (PF-ILD) Patients. NCT05139719 [65]	II	110	-Allocation: Randomized -Interventional Model: Parallel Assignment -Masking: Quadruple (Participant, Care Provider, Investigator, Outcomes Assessor)	Recruiting	Change from Baseline to Week 24 in FVC compared with placebo	HEC585 (yinfenidone)
Evaluating the Efficacy and Safety of HSK44459 in People With Idiopathic Pulmonary Fibrosis (IPF). NCT06764862 [69]	II	120	-Allocation: Randomized -Interventional Model: Parallel Assignment -Masking: Quadruple (Participant, Care Provider, Investigator, Outcomes Assessor)	Not yet recruiting	The change from baseline in FVC at week 12	HSK44459
Long-term Safety and Efficacy Study of HSK44459 Tablets in Patients With Idiopathic Pulmonary Fibrosis (IPF) NCT07019090 [70]	II	120	-Allocation: N/A -Interventional Model: Single Group Assignment -Masking: None (Open Label)	Not yet recruiting	The incidence and severity of adverse events during the study period	HSK4459
SB17170 Phase 2 Trial in IPF Patients. NCT06747923 [60]	II	30	-Allocation: Randomized -Interventional Model: Parallel Assignment -Masking: Double (Participant, Investigator)	Recruiting	Change from baseline in FVC	SB17170
WISPer: Evaluation of MTX-463 in Participants With Idiopathic Pulmonary Fibrosis (IPF). NCT06967805 [61]	II	164	-Allocation: Randomized -Interventional Model: Parallel Assignment -Masking: Quadruple (Participant, Care Provider, Investigator, Outcomes Assessor)	Recruiting	Change from baseline in FVC	MTX-463
A Study to Find Out Whether BI 765423 Has an Effect on Lung Function in People With Idiopathic Pulmonary Fibrosis (IPF) With or Without Standard Treatment. NCT07036523 [62]	II	71	-Allocation: Randomized -Interventional Model: Parallel Assignment -Masking: Quadruple (Participant, Care Provider, Investigator, Outcomes Assessor)	Recruiting	Absolute change from baseline in FVC at 12 weeks	BI765423
A Trial to Evaluate Efficacy and Safety of Buloxibutid in People With Idiopathic Pulmonary Fibrosis. (ASPIRE)NCT06588686 [63]	II	360	-Allocation: Randomized -Interventional Model: Parallel Assignment -Masking: Double (Participant, Investigator)	Recruiting	Evaluate the efficacy of buloxibutid compared to placebo in participants with IPF as assessed by FVC	Buloxibutid
A Phase 2 Study of CAL101 in Patients With Idiopathic Pulmonary Fibrosis (AURORA). NCT06736990 [64]	II	150	-Allocation: Randomized -Interventional Model: Parallel Assignment -Masking: Quadruple (Participant, Care Provider, Investigator, Outcomes Assessor)	Recruiting	Change from baseline in FVC compared to placebo	CAL101

**Table 2:** Signaling pathways, key targets, and their molecular functions in cellular regulation.

Drug	Target/Pathway	Mechanism of Action
Sufenidone (SC1011), HEC585	TGF-β/MAPK/TNF-α	Inhibits production of TGF-β, TNF-α and IL-1β; reduces fibroblast proliferation and collagen deposition
TDI01	RhoA/ROCK pathway	Selective Rho-kinase inhibitor → reduces myofibroblast contractility and ECM rigidity
Nerandomilast (BI 1015550), HSK44459	PDE4B/cAMP/TNF-α	PDE4B inhibitor → increases cAMP → anti-inflammatory & antifibrotic effect
BI 1819479, HRS-9813	Autotaxin/LPA pathway	Inhibits autotaxin → reduces LPA production and LPA-mediated fibroblast activation
Rentosertib (ISM001-055)	TNIK (Wnt/β-catenin pathway)	Inhibitor of TNIK → blocks Wnt signaling in fibroblasts
Ifetroban	TBXA2R (Thromboxane-prostanoid receptor)	TBXA2R antagonist → reduces TXA_2_-mediated fibroblast activation and vascular remodeling
Axatilimab	CSF1R → macrophage M2 pathway	Anti-CSF1R antibody → inhibits profibrotic M2 macrophages that produce TGF-β
Admilparant (BMS-986278)	LPA_1_ receptor (LPA pathway)	Oral LPA_1_ antagonist → inhibits LPA-mediated fibroblast activation and ECM deposition
ENV-101 (taladegib)	Smoothened (SMO)/Hedgehog signaling	Inhibits SMO, blocking Hedgehog pathway activation, reducing fibroblast activation and extracellular matrix deposition.
SB17170	HMGB1	Inhibits HMGB1-mediated signaling via RAGE/TLR4, reducing fibroblast activation and ECM deposition
MTX-463	WISP1/CCN4	Neutralizes WISP1, blocking pro-fibrotic signaling and fibroblast activation
BI765423	IL-11	Human monoclonal antibody; binds IL-11 and blocks its receptor-mediated pro-fibrotic signaling
Buloxibutid	Angiotensin II type 2 receptor AT2R	Activates AT2R to promote alveolar epithelial repair, reduce fibroblast activation, and limit extracellular matrix deposition
CAL101	S100A4/TLR4/TGF-β	Neutralizes extracellular S100A4 by blocking its receptor interactions, thereby inhibiting TLR4/NF-κB inflammatory signaling and S100A4-mediated amplification of TGF-β/SMAD signaling, with consequent anti-inflammatory and antifibrotic effects

## Data Availability

The data supporting the findings of this systematic review are available in the OSF registry: https://doi.org/10.17605/OSF.IO/YUVCA. The study selection process was documented according to the PRISMA guidelines. All data extracted are presented within the manuscript and are available from the corresponding author upon reasonable request. The datasets generated during and/or analyzed during the current study are available from the corresponding author upon reasonable request.
